# Association of *IL23R* Variants With Crohn’s Disease in Korean Children

**DOI:** 10.3389/fped.2019.00472

**Published:** 2019-11-19

**Authors:** Jeana Hong, Hye Ran Yang, Jin Soo Moon, Ju Young Chang, Jae Sung Ko

**Affiliations:** ^1^Department of Pediatrics, Kangwon National University School of Medicine, Chuncheon, South Korea; ^2^Department of Pediatrics, Seoul National University College of Medicine, Seoul, South Korea; ^3^Department of Pediatrics, Seoul National University Bundang Hospital, Seongnam-si, South Korea; ^4^Department of Pediatrics, SMG-SNU Boramae Medical Center, Seoul, South Korea

**Keywords:** Crohn's disease, single nucleotide polymorphisms, interleukin 23 receptor, genetics, children

## Abstract

**Background:** The interleukin 23 receptor gene (*IL23R*) is strongly associated with Crohn's disease (CD). It is unknown whether genetic variations in *IL23R* determine susceptibility for pediatric CD in Asian populations. Here, we investigated the association between *IL23R* variants and CD in Korean children.

**Methods:** Four single nucleotide polymorphisms (SNPs) of *IL23R* [rs76418789 (G149R), rs1004819, rs7517847, and rs1495965] were genotyped in 141 children with CD and 150 controls using DNA direct sequencing. The risk allele and genotype frequencies were compared between patients and controls. The association between clinical phenotypes and genotypes of patients was also analyzed.

**Results:** Two *IL23R* SNPs, rs76418789 (G149R), and rs1495965, were associated with CD in Korean pediatric patients as defense and risk loci, respectively. The odds ratio (OR) for rs76418789 (G149R) and rs1495965 was 0.409 (95% confidence interval [CI], 0.177–0.944; *p* = 0.031) and 1.484 (95% CI, 1.070–2.059; *p* = 0.018), respectively. Patients with the homozygous G allele of rs1495965 showed higher CD risk than those with other genotypes (GG vs. AA: OR, 2.256; 95% CI, 1.136–4.478; *p* = 0.019; GG vs. GA+AA: OR, 2.000; 95% CI, 1.175–3.404; *p* = 0.010). Additionally, they were more likely to have relatively invasive disease behavior of stenosis and/or penetration than simple inflammation (OR, 2.297; 95% CI, 1.065–4.950; *p* = 0.032).

**Conclusions:** This is the first study reporting *IL23R* variants in Asian pediatric patients with CD. *IL23R* was significantly associated with Korean pediatric CD, and the rs1495965 may influence the clinical features of CD in Korean children.

## Introduction

Crohn's disease (CD) is a type of inflammatory bowel disease (IBD) that causes chronic inflammation of the gastrointestinal tract. As a multifactorial disorder, a complex interaction of genetic, environmental, and immunological factors is implicated in the pathogenesis of IBD (1). Genetic or immunological factors are expected to play a more important role in developing CD at an earlier age because children have relatively short periods of exposure to environmental factors compared to adults (2). However, the exact pathogenesis of childhood-onset CD has not been elucidated.

The interleukin 23 (IL23)/T helper (Th) 17 pathway is suggested as an important immunological mechanism inducing chronic inflammatory responses in autoimmune diseases as well as IBD (3). Genome-wide association studies (GWAS) have revealed that IL23 receptor gene (*IL23R*) variants are strongly associated with CD (4). Additionally, a few studies reported that *IL23R* variants were associated with childhood-onset CD (5, 6). However, the reported associations were mostly based on studies in Western countries on Caucasian populations. The few case-control studies on Asian adults have been inconsistent in their conclusions (7–9). Moreover, the genetic influence of *IL23R* variants on the clinical phenotypes of CD was not determined even in a few Western studies targeting adults (10, 11).

Therefore, for the first time in Asian pediatric population, we aimed to identify *IL23R* polymorphisms to determine the association of *IL23R* variants with Korean children with CD. We also investigated the contribution of *IL23R* genotypes to the clinical features of pediatric CD.

## Materials and Methods

### Study Population

From January 2010 to December 2016, 150 patients with CD who were < 18 years old and treated at Seoul National University Children's Hospital, Seoul National University Bundang Hospital, and Kangwon National University Hospital were included in this study. The diagnosis of CD was based on clinical, endoscopic, radiological, and histological evaluations (12). Each patient's medical record was reviewed retrospectively and the collected data on clinical phenotypes were categorized according to the Paris classification for pediatric CD (13). For the analysis, the variables of the clinical phenotypes were binary-categorized based on sex, age at diagnosis, ileal involvement, upper GI tract involvement, and perianal disease. Disease behavior was also categorized as simple inflammation as well as stenosis, penetration, or both. For controls, 150 unrelated adults were selected from individuals who had undergone annual health checkups at the Seoul National University Hospital.

### Methods

We reviewed the literature and selected 4 single nucleotide polymorphisms (SNPs) in *IL23R* for genotyping. Three SNPs [rs76418789 (G149R), rs1004819, and rs1495965] were reported to be associated with CD in Korean adults, and 1 SNP [rs7517847] was reported as a protective locus in numerous studies on CD in Caucasian pediatric patients. We performed direct sequencing of the 4 SNPs using the genomic DNA samples to investigate their variants.

#### DNA Extraction

Genomic DNA was extracted from peripheral blood samples collected from the patients and the controls, using a HiGene genomic DNA prep kit (BIOFACT, Daejeon, Korea).

#### Polymerase Chain Reaction (PCR)

For PCR, all of the primers were designed using the Primer-3 website (http://bioinfo.ut.ee/primer3-0.4.0/). PCR was performed in a 25-μL reaction mixture, which consisted of 1.5 μL of each primer (10 pmol/μL), 15 μL of Solg 2× Raq PCR pre-mix (Solgent, Daejeon, Korea), and 100 ng of genomic DNA. The PCR conditions were as follows: 5 min of pre-denaturation at 95°C; followed by 34 cycles of 30 s denaturation at 95°C, 30 s of annealing at 63°C, and 1 min of elongation at 72°C; and a 5-min final extension at 72°C. The amplified DNA was separated by electrophoresis on 1.5% agarose gel and purified using the QIAquick PCR purification kit (Qiagen, Hilden, Germany).

#### Direct Sequencing

DNA sequencing of the PCR products was performed using an ABI Prism 3730XL analyzer (Applied Biosystems, Foster City, CA, USA).

### Statistical Analysis

The genotype frequencies were first evaluated for deviation from the Hardy-Weinberg equilibrium using the chi-squared test. Chi-squared analysis was used to evaluate the significance of the risk allele frequency (RAF) and genotype distribution between the case and the control groups. A logistic regression analysis was performed to investigate the association between variables and compute the *p*-values, odds ratios (ORs), and 95% confidence intervals (CIs). Haplotype analysis was performed using Haploview version 4.2 software (Broad Institute of the Massachusetts Institute of Technology and Harvard University, Cambridge, MA) (http://www.broadinstitute.org/haploview). All of the statistical analyses were performed using the PASW version 23.0 statistical software (Statistical Package for the Social Sciences [SPSS] Inc., Chicago, IL, USA) with a statistical significance level of *p* < 0.05.

## Results

### Clinical Features of Subjects

In total, 141 patients were included in the case group for the final analysis, excluding 9 patients whose genetic test results were incomplete, or whose medical records on clinical features were inaccurate. The case group consisted of 87 male and 54 female patients with the number of male patients 1.6 times higher than that of female patients. The clinical characteristics of the patients are presented in Table 1.

**Table 1:** Demographic characteristics and clinical features of patients (*n* = 141).

**Clinical characteristics**	**Patients, *n* (%)**
Male/female	87 (61.7%)/54 (38.3%)
Age at symptom onset (year)^*^	12.3 (10.4–14.0)
Age at diagnosis (year)^*^	12.9 (11.2–14.7)
A1a: 0–10 years	22 (15.6%)
A1b: 10–17 years	117 (83.0%)
A2: 17–40 years	2 (1.4%)
Duration of follow up (year)^*^	2.7 (1.1–4.8)
**Disease location**^†^	
L1: Ileum	13 (9.7%)
L2: Colon	13 (9.7%)
L3: Ileocolon	108 (80.6%)
L4: Upper GI disease^‡^	56 (42.7%)
L4a: esophagogastroduodenal disease	40 (30.5%)
L4b: jejunal/proximal ileal disease	10 (7.6%)
L4aL4b	6 (4.6%)
**Disease behavior**	
B1: non-stricturing, non-penetrating	103 (73.0%)
B2: stricturing	20 (14.2%)
B3: penetrating	14 (9.9%)
B2B3	4 (2.8%)
Perianal disease^§^	71 (50.4%)

*Clinical features are presented according to the Paris classification (13)*.

* *Data are expressed as the median (interquartile range)*.

† *Seven patients were excluded from analysis because of incomplete diagnostic workup for evaluation of the distal ileum (total, 134)*.

‡ *Ten patients were excluded from analysis because of missing information on the small bowel condition (total, 131)*.

§ *Perianal disease was defined as fistula, anal canal ulcer, or abscess*.

The correlation between each clinical phenotype and the demographic features of the patients was analyzed. The results showed that the clinical phenotype of CD differed according to the sex and age at diagnosis (Table 2). The risk of upper GI involvement (L4) was 3.1 times higher in male patients than in female patients (52.4% of males, 26.5% of females, *p* = 0.004). Moreover, male patients were 2.4 times more likely to have perianal disease than female patients (58.6% of males, 37.0% of females, *p* = 0.013). In addition, the risk of ileal involvement (L1 + L3) increased with age (*p* = 0.014), with a 4.8-fold increased risk in patients ≥ 10 years old at diagnosis compared to those who were < 10 years old at diagnosis (93.0% vs. 73.7%, *p* = 0.008).

**Table 2:** Association between demographic characteristics of patients and disease features according to the Paris classification (13).

**Disease location/behavior**	**Male/female, *n* (%)**	OR^*^ **(95% CI)**	***p-*value**	**Age at diagnosis**		OR^*^ **(95% CI)**	***p-*value**
				**<10 years (A1a)**	**≥10 years (A1b + A2)**		
Ileal involvement (L1 + L3)^†^	76 (90.5%)/45 (90%)	1.056 (0.325–3.424)	0.928	14 (73.7%)	107 (93.0%)	4.777 (1.371–16.648)	0.008
Upper GI disease (L4)^‡^	43 (52.4%)/13 (26.5%)	3.053 (1.416–6.582)	0.004	9 (47.4%)	47 (42.0%)	0.803 (0.303–2.131)	0.660
Stricturing and/or penetrating (B2 + B3 + B2B3)	21 (24.1%)/17 (31.5%)	0.693 (0.325–1.474)	0.339	8 (36.4%)	30 (25.2%)	0.590 (0.225–1.544)	0.279
Perianal disease^§^	51 (58.6%)/20 (37.0%)	2.408 (1.198–4.840)	0.013	9 (40.9%)	62 (52.1%)	1.571 (0.624–3.954)	0.335

*OR, odds ratio; CI, confidence interval*.

* *Reference is female or < 10 years of age*.

† *Seven patients were excluded from analysis because of incomplete diagnostic workup for evaluation of the distal ileum (total, 134)*.

‡ *Ten patients were excluded from analysis because of missing information on the small bowel condition (total, 131)*.

§ *Perianal disease was defined as fistula, anal canal ulcer, or abscess*.

### Association of *IL23R* Polymorphisms With CD Susceptibility

Four *IL23R* SNPs were analyzed in 141 patients with CD and 150 healthy controls. No significant deviation from the Hardy-Weinberg equilibrium was observed in the study population. The RAFs of the cases and controls are described in Table 3. Among the 4 SNPs, 2 *IL23R* variants, rs76418789 (G149R) and rs1495965, showed an association with CD. The risk allele A of rs76418789 (G149R) exhibited a lower frequency in patients with CD (OR, 0.409; 95% CI, 0.177–0.944; *p* = 0.031). In addition, the frequency of the G allele of rs1495965 was higher in patients with CD than in controls (OR, 1.484; 95% CI, 1.070–2.059; *p* = 0.018).

**Table 3:** Allele frequency of patients with Crohn's disease and controls.

**SNP IDs**	**Location**	**Allele**		**Risk allele frequency**		**HWE** ***p*-value**	**OR** **(95% CI)**	***p*-value**
		1^*^	**2**	**CD**	**Control**			
rs76418789 (G149R)	Exon 4	A	G	8/282 = 0.028	20/300 = 0.067	0.662	0.409 (0.177–0.944)	0.031
rs1004819	Intron 5	T	C	164/282 = 0.582	166/300 = 0.553	0.981	1.122 (0.808–1.558)	0.492
rs7517847	Intron 6	G	T	112/282 = 0.397	121/300 = 0.403	0.248	0.975 (0.699–1.358)	0.879
rs1495965	Intergenic	G	A	163/282 = 0.578	144/300 = 0.480	0.136	1.484 (1.070–2.059)	0.018

* *Allele 1 is a risk allele; OR, odds ratio; HWE, Hardy-Weinberg equilibrium; CI, confidence interval; NA, not applicable*.

The genotype distribution of the 4 SNPs in the case and control groups is shown in Table 4. The association of the genotypes with CD was analyzed with the genotype frequencies reconstructed as a genetic model (dominant, recessive). In the dominant model, the genetic variant of rs76418789 (G149R) showed a 0.4-fold lower risk of CD when it had at least one risk allele A than when it had homozygous wild-type alleles (OR, 0.415; 95% CI, 0.175–0.981; *p* = 0.040). For rs1495965, subjects with homozygote of the risk allele G exhibited a two-fold higher risk of CD than the subjects with the other genotypes (GG vs. AA: OR, 2.256; 95% CI, 1.136–4.478; *p* = 0.019; GG vs. GA + AA: OR, 2.000; 95% CI, 1.175–3.404; *p* = 0.010).

**Table 4:** Comparison of genotype distribution between patients with Crohn's disease and controls.

**SNP IDs**	**Allele**		**Genotype of case**				**Genotype of controls**				**11 vs. 22**		**Dominant model** **(11+12 vs. 22)**		**Recessive model** **(11 vs. 12+22)**	
	1^*^	**2**	**11**	**12**	**22**	**Total**	**11**	**12**	**22**	**Total**	**OR** **(95% CI)**	***p-*value**	**OR** **(95% CI)**	***p-*value**	**OR** **(95% CI)**	***p-*value**
rs76418789 (G149R)	A	G	0 (0%)	8 (5.7%)	133 (94.3%)	141 (100%)	1 (0.7%)	18 (12.0%)	131 (87.2%)	150 (100%)	NA	NA	0.415 (0.175–0.981)	0.040	NA	NA
rs1004819	T	C	54 (38.1%)	56 (40.0%)	31 (21.9%)	141 (100%)	46 (30.7%)	74 (49.6%)	30 (19.7%)	150 (100%)	1.136 (0.600–2.149)	0.695	0.887 (0.504–1.560)	0.677	1.406 (0.406–2.354)	0.194
rs7517847	G	T	23 (16.4%)	66 (46.4%)	52 (37.1%)	141 (100%)	21 (14.2%)	79 (52.8%)	50 (33.1%)	150 (100%)	1.053 (0.519–2.137)	0.886	0.856 (0.529–1.386)	0.526	1.197 (0.630–2.275)	0.582
rs1495965	G	A	47 (34.0%)	69 (48.7%)	25 (17.3%)	141 (100%)	30 (20.0%)	84 (56.0%)	36 (24.0%)	150 (100%)	2.256 (1.136–4.478)	0.019	1.465 (0.827–2.596)	0.189	2.000 (1.175–3.404)	0.010

* *Allele 1 is a risk allele; OR, odds ratio; CI, confidence interval; NA, not applicable*.

### Haplotype Analysis of *IL23R* Polymorphisms

**Figure 1:**
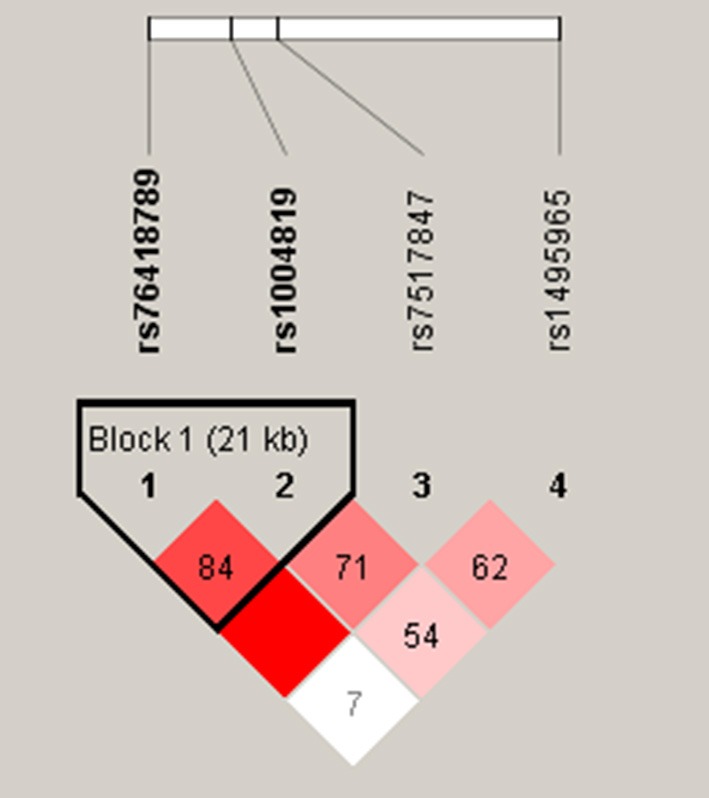
Linkage disequilibrium (LD) and haplotype block structures of *IL23R*. Single nucleotide polymorphisms, rs76418789 (G149R) and rs1004819, in tight LD were organized in a single haplotype block.

The pairwise linkage disequilibrium (LD) was checked with the 4 analyzed SNPs and the LD block is shown in a Figure 1. A single LD block harbored both rs76418789 (G149R) and rs1004819. Among the 3 haplotypes whose frequency was > 1% of the 4 possible ones, there was no statistically significant association between these haplotypes and CD.

### Genotype and Phenotype Analysis

The correlation between the clinical features and the genotypes of the SNPs was analyzed in patients with CD. After adjustment for sex and age at diagnosis, only rs1495965 revealed to be associated with clinical phenotypes (Table 5). The patients with a homozygous variant of the G allele showed a more severe disease pattern of stenosis and/or penetration than simple inflammation, compared to patients with the heterozygous variants of the G allele or wild-type (OR, 2.297; 95% CI, 1.065–4.950; *p* = 0.032). Patients with the homozygous G allele exhibited a 6-fold higher likelihood of ileal involvement than those with heterozygous variants or wild-type allele, although the difference was not statistically significant (OR, 6.103; 95% CI, 0.755–49.336; *p* = 0.090).

**Table 5:** Association between rs1495965 genotypes and phenotypes in patients with Crohn's disease.

**Clinical Characteristics**	**GG**	**GA**	**AA**	***p*-value**	**GG**	**GA + AA**	**OR**	***p*-value**
	**(*n* = 47)**	**(*n* = 69)**	**(*n* = 25)**		**(*n* = 47)**	**(*n* = 94)**	**(95% CI)**	
Male/female	29/18	43/26	15/10	0.979	29/18	58/36	1.000 (0.487–2.055)	1.000
Age at symptom onset (year)^*^	12.3 (11.2–14.1)	11.8 (9.6–13.7)	12.9 (10.2–14.5)	0.325	12.3 (11.2–14.1)	12.3 (10.2–13.9)		0.280
**Age at diagnosis**								
<10 years (A1a)	5 (10.6%)	14 (20.3%)	3 (12.0%)	0.320	5 (10.6%)	17 (18.1%)	Ref.	
≥10 years (A1b+A2)	42 (89.4%)	55 (79.7%)	22 (88.0%)		42 (89.4%)	77 (81.9%)	1.855 (0.639–5.383)	0.251
**Disease location**								
Ileal involvement (L1 + L3)^†^	44 (97.8%)	57 (87.7%)	20 (83.3%)	0.095	44 (97.8%)	77 (86.5%)	6.103 (0.755–49.336)	0.090
upper GI disease (L4)^‡^	20 (43.5%)	27 (41.5%)	9 (45.0%)	0.956	20 (43.5%)	36 (42.4%)	1.070 (0.503–2.279)	0.860
**Disease behavior**								
Non-stricturing, non-penetrating (B1)	29 (61.7%)	54 (78.3%)	20 (80.0%)	0.098	29 (61.7%)	74 (78.7%)	Ref.	
Stricturing and/or penetrating (B2 + B3 + B2B3)	18 (38.3%)	15 (21.7%)	5 (20.0%)		18 (38.3%)	20 (21.3%)	2.297 (1.065–4.950)	0.032
Perianal disease^§^	23 (48.9%)	34 (49.3%)	13 (52.0%)	0.966	23 (48.9%)	47 (50.0%)	0.887 0.431–1.825	0.745

*OR, odds ratio; CI, confidence interval*.

* *Data are expressed as the median (interquartile range)*.

† *Seven patients were excluded from analysis because of incomplete diagnostic workup for evaluation of the distal ileum (total, 134)*.

‡ *Ten patients were excluded from analysis because of missing information on the small bowel condition (total, 131)*.

§ *Perianal disease was defined as fistula, anal canal ulcer, or abscess*.

## Discussion

This study showed that two of the *IL23R* variants, rs76418789 (G149R) and rs1495965, were associated with CD susceptibility in Korean pediatric patients. We found a correlation between the homozygous G allele of rs1495965 and the phenotype with more invasive disease behavior in Korean children with CD.

The rs76418789 (G149R) was the first identified locus in a Chinese study where the genotypes revealed no significant association with 50 Chinese patients with CD (14). In recent studies on Korean adult patients with IBD, rs76418789 (G149R) showed a CD-associated gene locus (15, 16). A subsequent GWAS with Korean adults confirmed rs76418789 (G149R) as a significant factor protecting against CD in Koreans (17). In addition, a study of 176 Japanese adults with CD reported allele A as significantly protective against CD, which was consistent with the findings of Korean studies (18). In Western populations, there have been few studies regarding the association of rs76418789 (G149R) with CD (19). According to the 1000 Genomes Project (https://www.ncbi.nlm.nih.gov/variation/tools/1000genomes/), the risk allele frequency of rs76418789 (G149R) is 0.0112, which is 4–5 times less when compared to East Asian controls of the previous studies (14–17). It means that rs76418789 (G149R) is monomorphic in most European populations. Therefore, rs76418789 (G149R) is suggested to be an ethnic group-specific SNP and our results strongly supports the previous results of protective role of rs76478789 in CD pathogenesis. However, further studies on rs76418789 (G149R) variants are needed to confirm the trend in Western patients with CD. There have been several studies of the association of rs1004819 with CD susceptibility in Asian populations. In studies of Japanese, Chinese, and Malaysian populations, there was no association of rs1004819 with CD (7–9). However, a study of 380 Korean adults with CD reported an association with rs1004819 (20). Although the genotype distribution of the controls was consistent with our results, the Korean adults study reported a statistically significant association with an odds ratio of the risk allele as low as 1.262, whereas our study showed no association. As a result, we suggest that the rs1004819 is less likely to be a risk locus, at least for early-onset CD in Korean patients.

rs7517847 has been consistently reported to show strong associations with CD as a protective factor in Caucasians (21). The G allele of rs7517847 has been reported as a protective allele in Canadian pediatric patients (5). Currently, the only study targeting Asian ethnic groups was performed in Japanese adults with CD (7). In contrast to studies performed in Western countries, no association between the rs7517847 SNP and CD in Japanese adults with CD has been reported. We, for the first time, analyzed the rs7517847 genotype targeting the Korean ethnic group with CD, and the risk allele frequency of G was reduced among patients, although the difference was not statistically significant. Finally, our study predicted no association between rs7517847 and CD in Asian patients. However, further studies are required for verification of the results in Asians with CD including adults.

A recent study of Korean adults reported that the G allele of rs1495965 in the dominant genetic model was significantly associated with CD (20). Our present results are consistent with the findings of the study. In contrast, a Japanese study showed no association between this SNP and 484 adults with CD (7). Because there was no significant difference in the risk allele frequency between both controls of Japanese and Korean adults, the genotype differences in patient populations could have been caused by the ethnic difference in CD development. Our results suggest that rs1495965 may be a Korean-specific locus whose G allele could be a risk allele in Korean patients with CD. Further cohorts for replication are needed in Asian population to confirm the association.

A recent meta-analysis of over 60 case-control association studies reported a significant association between *IL23R* and Caucasians with CD, but not Asian patients (21). However, the meta-analysis included only a few studies on Asian patients, excluding recent studies on Korean adults. We summarize the reported Asian studies for the association of *IL23R* with CD susceptibility in Table 6. Most of the studies targeted adult patients, except a few studies including some children under the age of 16 years. It is thus notable that this study was conducted in such a large number of 141 children with CD as a single ethnic group in an Asian population.

**Table 6:** Summary of Asian studies for the association of *IL23R* with Crohn's disease (CD) susceptibility (7–9, 14–16, 18, 20).

**SNP IDs**	**Allele**		**Ethnicity**	**No. of subjects**		**OR (95% CI)**	***p*-value**	**Association**	**References**
	1^*^	**2**		**CD (%)^†^**	**Control**				
rs76418789 (G149R)	A	G	Chinese	50 (7.4%)	50	2.042 (0.182–22.889)	0.621	No	(14)
			South Korean	201 (NR)	258	0.335 (0.158–0.709)	0.003	Yes	(15)
			South Korean	500 (NR)	1000	0.470 (0.36–0.61)	<0.001	Yes	(16)
			Japanese	176 (0%)	358	0.222 (0.100–0.491)	<0.001	Yes	(18)
rs1004819	T	C	Japanese	482 (NR)	439	1.052 (0.875–1.265)	0.587	No	(7)
			South Korean	380 (13.9%)	379	1.262 (1.028–1.548)	0.026	Yes	(20)
			Malaysian	80 (NR)	100	1.016 (0.659–1.588)	0.941	No	(8)
			Chinese	420 (7.1%)	500	0.930 (0.83–1.03)	0.180	No	(9)
rs7517847	G	T	Japanese	484 (NR)	438	1.158 (0.962–1.393)	0.120	No	(7)
rs1495965	G	A	Japanese	484 (NR)	437	1.120 (0.933–1.346)	0.224	No	(7)
			South Korean	380 (13.9%)	377	1.310 (1.070–1.603)	0.009	Yes	(20)
			Chinese	420 (7.1%)	500	1.020 (0.930–1.110)	0.740	No	(9)

* Allele 1 is a risk allele;

† *Proportion of the patients ≤16 years in subjects; NR, not reported; OR, odds ratio for risk allele frequency; CI, confidence interval*.

There have been several studies to determine the correlation between clinical phenotypes of CD and the associated genes such as *NOD2/CARD15, ATG16L1*, and *TNFSF15* (22–25). Most of these studies were performed in Western countries targeting adult patients, and the results were inconsistent with their conclusion. Regarding the SNPs of *Il23R*, rs1004819 was associated with the ileal disease phenotype of CD in a German study. Meanwhile, rs11209026 (Arg381Gln) showed no sub-phenotype association in adult CD in a British study (10, 11). The present study investigated the association between the genotypes and clinical features of CD in pediatric patients categorized according to the Paris classification (13). As previously reported, we also found that the sex and age at diagnosis were associated with clinical CD phenotypes (26). After adjusting for sex and age variables, we discovered that the homozygous risk allele G of rs1495965 was more closely associated with the invasive disease behavior of stenosis or penetration than with simple inflammation. Our study provide evidence that the genotype of rs1495965 could be a marker for predicting serious clinical courses in children and indicates the need for further investigation of the utility of the genotypes as a predictive marker.

Compared with 11 GWAS on adult IBD, 2 GWAS on childhood-onset CD have demonstrated that the genotypes of early and adult-onset CD differ genetically, revealing 7 new gene loci that were not found in adults (27–29). Genetic susceptibility could therefore be suggested as an important factor for childhood-onset CD. However, several reports have failed to reach a consensus on the association between childhood-onset CD and the loci of various genes including *IL23R* (30, 31). Assuming that early presentation of the disease is one of the phenotypes of CD, a subsequent study on the same gene loci identified in the previous adult CD studies in a pediatric population with the same ethnicity, similar to the present study, would be particularly significant. Simultaneous comparative analysis of specific genes in adult and pediatric patients could identify genetic determinants of the pathogenesis of childhood-onset CD. Notably, the present results showed that 2 of the 3 SNPs that had a significant association with Korean adult CD also carried an association with Korean pediatric CD, suggesting that these 2 loci may be candidates for childhood-onset CD in Koreans.

Our study had some limitations. First, a functional analysis of *IL23R* was not performed. Although the investigated SNPs of *IL23R* were found to be associated with CD risk, the functions of the variants are yet to be elucidated. As a non-synonymous SNP, the coding variant rs76418789 (G149R) in exon 4 changes the glycine to arginine at position 149, affecting highly conserved residues in the extracellular domain of the receptor (19). To predict the effects of the change in sequence on the variant's function, we used a sequence homology-based tool, Sorting Intolerant From Tolerant (SIFT), which predicted the change to be “deleterious” (32). The intronic polymorphisms, rs1004819 and rs7517847, might influence the regulation of differential splicing (21). Second, healthy adults were selected as the control group in this study. However, given the time it takes for the disease to manifest in genetically susceptible individuals, it would be more appropriate to select those adults for the control group who have not had the disease for more than one generation. Additionally, we matched the ethnicity of the adult controls to Koreans for our pediatric study. In fact, there are several published studies in which children with CD have been compared with healthy-adult controls (30, 33).

In conclusion, this is the first study on an Asian population identifying the association between *IL23R* variants and childhood-onset CD. The rs76418789 (G149R) and rs1495965 variants may be candidate loci for childhood-onset CD in Koreans. Furthermore, the homozygous risk allele of rs1495965 could be suggested as a predictive marker for relatively severe disease behavior.

## Data Availability Statement

All datasets analyzed for this study are included in the article.

## Ethics Statement

The study was approved by the Institutional Review Board of the Seoul National University Hospital, Seoul, Korea (IRB No. H-1311-044-533). Written informed consent was obtained from all participating patients, their legal guardians, or both.

## Author Contributions

Conceptualization: JH, JM, and JK. Data curation: JH, HY, JM, and JK. Formal analysis, writing—review, and editing: JH, JC, and JK. Writing—original draft: JH.

### Conflict of Interest

The authors declare that the research was conducted in the absence of any commercial or financial relationships that could be construed as a potential conflict of interest.

## References

[1] Kaser A Zeissig S Blumberg RS. Inflammatory bowel disease. Annu Rev Immunol. (2010) 28:573–621. 10.1146/annurev-immunol-030409-101225 20192811 PMC4620040

[2] de Ridder L Weersma RK Dijkstra G van der Steege G Benninga MA Nolte IM. Genetic susceptibility has a more important role in pediatric-onset Crohn's disease than in adult-onset Crohn's disease. Inflamm Bowel Dis. (2007) 13:1083–92. 10.1002/ibd.20171 17476680

[3] Lees CW Barrett JC Parkes M Satsangi J. New IBD genetics: common pathways with other diseases. Gut. (2011) 60:1739–53. 10.1136/gut.2009.199679 21300624

[4] Duerr RH Taylor KD Brant SR Rioux JD Silverberg MS Daly MJ. A genome-wide association study identifies IL23R as an inflammatory bowel disease gene. Science. (2006) 314:1461–3. 10.1126/science.1135245 17068223 PMC4410764

[5] Amre DK Mack D Israel D Morgan K Lambrette P Law L. Association between genetic variants in the *IL-23R* gene and early-onset Crohn's disease: results from a case-control and family-based study among Canadian children. Am J Gastroenterol. (2008) 103:615–20. 10.1111/j.1572-0241.2007.01661.x 18047539

[6] Van Limbergen J Russell RK Nimmo ER Drummond HE Smith L Davies G. IL23R Arg381Gln is associated with childhood onset inflammatory bowel disease in Scotland. Gut. (2007) 56:1173–4. 10.1136/gut.2007.122069 17337463 PMC1955485

[7] Yamazaki K Onouchi Y Takazoe M Kubo M Nakamura Y Hata A. Association analysis of genetic variants in *IL23R, ATG16L1* and 5p13.1 loci with Crohn's disease in Japanese patients. J Hum Genet. (2007) 52:575–83. 10.1007/s10038-007-0156-z 17534574

[8] Chua KH Hilmi I Lian LH Patmanathan SN Hoe SZ Lee WS. Association between inflammatory bowel disease gene 5 (*IBD5*) and interleukin-23 receptor (*IL23R*) genetic polymorphisms in Malaysian patients with Crohn's disease. J Dig Dis. (2012) 13:459–65. 10.1111/j.1751-2980.2012.00617.x 22908971

[9] Zhang J Chen J Gu J Guo H Chen W. Association of IL23R and ATG16L1 with susceptibility of Crohn's disease in Chinese population. Scand J Gastroenterol. (2014) 49:1201–6. 10.3109/00365521.2014.936031 25048429

[10] Glas J Seiderer J Wetzke M Konrad A Torok HP Schmechel S. rs1004819 is the main disease-associated IL23R variant in German Crohn's disease patients: combined analysis of IL23R, CARD15, and OCTN1/2 variants. PLoS ONE. (2007) 2:e819. 10.1371/journal.pone.0000819 17786191 PMC1950565

[11] Tremelling M Cummings F Fisher SA Mansfield J Gwilliam R Keniry A IL23R variation determines susceptibility but not disease phenotype in inflammatory bowel disease. Gastroenterology. (2007) 132:1657–64. 10.1053/j.gastro.2007.02.051 17484863 PMC2696256

[12] IBD Working Group of the European Society for Paediatric Gastroenterology Hepatology and Nutirition Inflammatory bowel disease in children and adolescents: recommendations for diagnosis–the Porto criteria. J Pediatr Gastroenterol Nutr. (2005) 41:1–7. 10.1097/01.mpg.0000163736.30261.82 15990620

[13] Levine A Griffiths A Markowitz J Wilson DC Turner D Russell RK. Pediatric modification of the Montreal classification for inflammatory bowel disease: the Paris classification. Inflamm Bowel Dis. (2011) 17:1314–21. 10.1002/ibd.21493 21560194

[14] Bin C Zhirong Z Xiaoqin W Minhu C Mei L Xiang G. Contribution of rs11465788 in IL23R gene to Crohn's disease susceptibility and phenotype in Chinese population. J Genet. (2009) 88:191–6. 10.1007/s12041-009-0027-9 19700857

[15] Kim SW Kim ES Moon CM Park JJ Kim TI Kim WH. Genetic polymorphisms of IL-23R and IL-17A and novel insights into their associations with inflammatory bowel disease. Gut. (2011) 60:1527–36. 10.1136/gut.2011.238477 21672939

[16] Hong SN Park C Park SJ Lee CK Ye BD Kim YS. Deep resequencing of 131 Crohn's disease associated genes in pooled DNA confirmed three reported variants and identified eight novel variants. Gut. (2016) 65:788–96. 10.1136/gutjnl-2014-308617 25731871

[17] Yang SK Hong M Zhao W Jung Y Baek J Tayebi N. Genome-wide association study of Crohn's disease in Koreans revealed three new susceptibility loci and common attributes of genetic susceptibility across ethnic populations. Gut. (2014) 63:80–7. 10.1136/gutjnl-2013-305193 23850713

[18] Onodera K Arimura Y Isshiki H Kawakami K Nagaishi K Yamashita K. Low-frequency IL23R coding variant associated with Crohn's disease susceptibility in Japanese subjects identified by personal genomics analysis. PLoS ONE. (2015) 10:e0137801. 10.1371/journal.pone.0137801 26375822 PMC4574159

[19] Momozawa Y Mni M Nakamura K Coppieters W Almer S Amininejad L. Resequencing of positional candidates identifies low frequency IL23R coding variants protecting against inflammatory bowel disease. Nat Genet. (2011) 43:43–7. 10.1038/ng.733 21151126

[20] Yang SK Park M Lim J Park SH Ye BD Lee I Contribution of IL23R but not ATG16L1 to Crohn's disease susceptibility in Koreans. Inflamm Bowel Dis. (2009) 15:1385–90. 10.1002/ibd.20921 19334001

[21] Xu WD Xie QB Zhao Y Liu Y. Association of Interleukin-23 receptor gene polymorphisms with susceptibility to Crohn's disease: a meta-analysis. Sci Rep. (2015) 5:18584. 10.1038/srep18584 26678098 PMC4683513

[22] Economou M Trikalinos TA Loizou KT Tsianos EV Ioannidis JP. Differential effects of NOD2 variants on Crohn's disease risk and phenotype in diverse populations: a metaanalysis. Am J Gastroenterol. (2004) 99:2393–404. 10.1111/j.1572-0241.2004.40304.x 15571588

[23] Vermeire S Wild G Kocher K Cousineau J Dufresne L Bitton A. CARD15 genetic variation in a Quebec population: prevalence, genotype-phenotype relationship, and haplotype structure. Am J Hum Genet. (2002) 71:74–83. 10.1086/341124 12019468 PMC384994

[24] Yang DH Yang SK Song K Hong M Park SH Lee HS. *TNFSF15* is an independent predictor for the development of Crohn's disease-related complications in Koreans. J Crohns Colitis. (2014) 8:1315–26. 10.1016/j.crohns.2014.04.002 24835165

[25] Prescott NJ Fisher SA Franke A Hampe J Onnie CM Soars D. A nonsynonymous SNP in ATG16L1 predisposes to ileal Crohn's disease and is independent of CARD15 and IBD5. Gastroenterology. (2007) 132:1665–71. 10.1053/j.gastro.2007.03.034 17484864

[26] de Bie CI Paerregaard A Kolacek S Ruemmele FM Koletzko S Fell JM. Disease phenotype at diagnosis in pediatric Crohn's disease: 5-year analyses of the EUROKIDS Registry. Inflamm Bowel Dis. (2013) 19:378–85. 10.1002/ibd.23008 22573581

[27] Imielinski M Baldassano RN Griffiths A Russell RK Annese V Dubinsky M. Common variants at five new loci associated with early-onset inflammatory bowel disease. Nat Genet. (2009) 41:1335–40. 10.1038/ng.489 19915574 PMC3267927

[28] Kugathasan S Baldassano RN Bradfield JP Sleiman PM Imielinski M Guthery SL. Loci on 20q13 and 21q22 are associated with pediatric-onset inflammatory bowel disease. Nat Genet. (2008) 40:1211–5. 10.1038/ng.203 18758464 PMC2770437

[29] Lees CW Satsangi J. Genetics of inflammatory bowel disease: implications for disease pathogenesis and natural history. Expert Rev Gastroenterol Hepatol. (2009) 3:513–34. 10.1586/egh.09.45 19817673

[30] Essers JB Lee JJ Kugathasan S Stevens CR Grand RJ Daly MJ Established genetic risk factors do not distinguish early and later onset Crohn's disease. Inflamm Bowel Dis. (2009) 15:1508–14. 10.1002/ibd.20922 19322901 PMC2768775

[31] Henderson P van Limbergen JE Wilson DC Satsangi J Russell RK. Genetics of childhood-onset inflammatory bowel disease. Inflamm Bowel Dis. (2011) 17:346–61. 10.1002/ibd.21283 20839313

[32] Vaser R Adusumalli S Leng SN Sikic M Ng PC. SIFT missense predictions for genomes. Nat Protoc. (2016) 11:1–9. 10.1038/nprot.2015.123 26633127

[33] Lee YJ Kim KM Jang JY Song K. Association of TNFSF15 polymorphisms in Korean children with Crohn's disease. Pediatr Int. (2015) 57:1149–53. 10.1111/ped.12686 25998826

